# Accessible versatility underpins the deep evolution of plant specialized metabolism

**DOI:** 10.1007/s11101-023-09863-2

**Published:** 2023-03-30

**Authors:** Armin Dadras, Tim P. Rieseberg, Jaccoline M. S. Zegers, Janine M. R. Fürst-Jansen, Iker Irisarri, Jan de Vries, Sophie de Vries

**Affiliations:** 1 Department of Applied Bioinformatics, University of Goettingen, Institute for Microbiology and Genetics, Goldschmidtstr. 1, 37077 Göttingen, Germany; 2 University of Goettingen, Campus Institute Data Science (CIDAS), Goldschmidtstr. 1, 37077 Göttingen, Germany; 3 Department of Applied Bioinformatics, University of Goettingen, Goettingen Center for Molecular Biosciences (GZMB), Goldschmidtstr. 1, 37077 Göttingen, Germany

**Keywords:** Plant Terrestrialization, Specialized Metabolism, Land Plants, Streptophytes, Evolutionary Biochemistry, Evolutionary Metabolomics

## Abstract

The evolution of several hallmark traits of land plants is underpinned by phytochemical innovations. The specialized metabolism of plants can appear like a teeming chaos that has yielded an ungraspable array of chemodiversity. Yet, this diversity is the result of evolutionary processes including neutral evolution, drift, and selection that have shaped the metabolomic networks. Deciphering the evolutionary history of the specialized metabolome in the context of plant terrestrialization has only just begun. Studies on phytochemistry of model organisms and crop plants enabled the sketch of a blueprint for the biochemical landscape of land plants and a good idea on the diversity that can be explored. Evolutionary metabolomics has in the past been successfully used to identify traits that were critical for domestication of angiosperms or to unravel key innovations in land plants. Owing to recent advances in the study of non-model land plants and their close streptophyte algal relatives we can now begin to appreciate the variation of metabolic networks across the green lineage—and understand convergent solutions to similar environmental challenges and effects that plant terrestrialization had on these networks. Here, we highlight the significant progress made with regard to identifying metabolomic diversity by adding non-model organisms to the equation. We discuss the role of neutral evolution in the context of metabolomic diversity and the effects that environmental challenges had on the lineage-specific specialized metabolism from an evolutionary point of view.

## Introduction

**Fig. 1:**
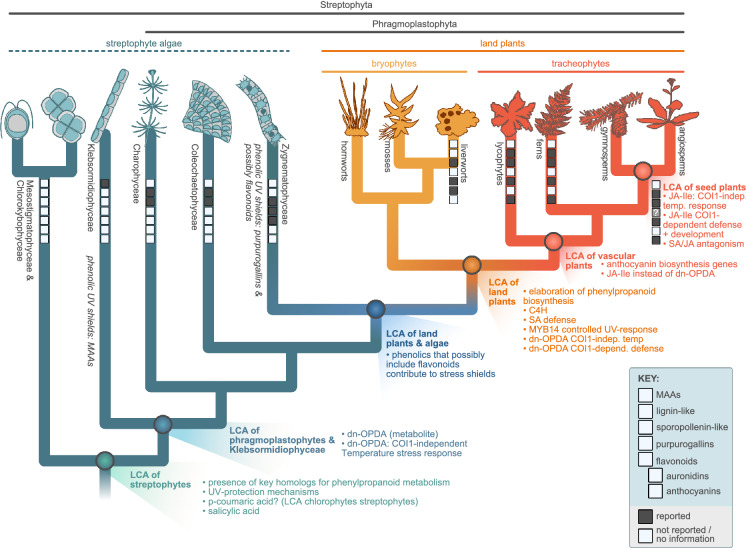
Evolutionary dynamics in key biochemical pathways of streptophytes. A cladogram of streptophyte diversity. Nodes represent last common ancestors (LCAs) and highlight the building blocks for dynamic evolutionary processes that gave rise to important specialized metabolic pathways that are discussed in this manuscript. Compounds included in this figure are shown in the key, auronidins and anthocyanins are indented within the key because they derive from the flavonoid pathway. For mapping on the tree indentation is removed for better visualisation. Filled boxes indicate that a compound/feature has been reported in the literature; white/unfilled boxes indicate that, to our knowledge, there is no such report—it does not mean outright absence. JA-Ile: jasmonoyl–isoleucine, COI1: coronatine-insensitive 1, SA: salicylic acid, JA: jasmonic acid, C4H: cinnamate 4-hydroxylase, dn-OPDA: dinor-12-oxo-phytodienoic acid

Land plants are characterized by a series of unique traits. Some of these traits are critical for living on land, having provided embryophytes during the infancy of their lineage with selective advantages in the terrestrial habitat. Among these have been several structural and physiological innovations, e.g., protection against water loss aided by stomata (Raven 2002; Harris et al. 2020), and molecular and physiological responses to terrestrial stressors, such as rapid and drastic shifts in temperature and irradiance and water scarcity (de Vries and Archibald 2018; Fürst-Jansen et al. 2020). With regard to the latter, several phytochemical traits, such as the ability to produce UV-protectant metabolites and the synthesis of phytohormones integral for physiological response to stress, have been highlighted (de Vries et al. 2018a, 2018b; Han 2019; Fürst-Jansen et al. 2020; Maeda and Fernie 2021). That said, the immediate adaptive advantage of any of these traits upon their first appearance might be questioned. According to evolutionary theory, traits arose by chance in a given last common ancestor (LCA) and the fact that we can observe them today means that at that time they were not detrimental or had severe fitness costs. Some traits might have been adaptive from the beginning or became adaptive later on, but many other traits have simply tagged along—evolving neutrally, being the result of non-adaptive processes—and/or evolutionary (historical) contingency. When organisms, in which these traits occur, encountered a given environmental condition for longer or recurring periods of time, some of the (metabolic) traits that tagged along could have offered a selective advantage. From a metabolic perspective, we might think of neutrally evolving metabolic networks that diversify and provide building blocks for natural selection to act upon. In this sense, a species’ metabolic repertoire can be seen as the consequence of the evolution of its genome, which is governed by its own laws of organization, including whole genome or segmental duplications, gene loss or regulatory constraints, or insertions of transposable elements. All these events, which often have little to do with metabolism, are important players in determining what is metabolically possible. How new metabolic routes, variation and diversity emerged during plant terrestrialization and land plant evolution and which convergent solutions have arisen by chance is the main focus of this review. We illustrate the deep origins of diverse networks of specialized metabolism that have an adaptive component to it (Fig. 1) with regard to the evolution of the green lineage and discuss the evolutionary processes that determined the metabolic diversity that we see in today's extant species.

## Challenges in the evolutionary analyses of complex plant specialized metabolism

Metabolomics, genomics, and functional studies in model organisms have greatly contributed to our understanding of metabolic pathways of plants—or any metabolic network in general. To study the evolution of metabolic networks, we contextualize presence/absence information from genomic and metabolomic data across evolutionary distinct lineages with phylogenetic information. These networks are defined by functional studies in model species such as *Arabidopsis thaliana*: When we identify orthologous genes in different species, we presume that they perform similar reactions; when we measure similar compounds in different species, we presume that these are produced by orthologous sets of genes or networks. This conformism aligns well with the concept that similar traits are most likely due to common descent. Simultaneously, this approach is often conservative, viewing metabolic networks from the standpoint of a model species; it favors the “knowns” and does not accommodate putative metabolomic complexity present in a phylodiverse set of species. As a consequence, discoveries of new metabolic routes in non-model organisms produce the same category of metabolites as the model species tend to be considered a variant of the canonical pathway. What is canonical and what is a variant is often also determined by the historical order of discovery. Especially for the hyperdiverse specialized metabolism, these assumptions may be misleading and hampering our interpretations on not only evolutionary history of metabolic pathways but also our interpretation of what the key traits might have been for certain happenings. The phenylpropanoid pathway (PPP) contributes to various compounds that offer aid against terrestrial stressors and are characteristic of land plants (Vogt 2010). But are they characteristic for land plants? Lignin-like compounds have been reported from the closest algal relatives of land plants, the streptophyte algae (Delwiche et al. 1989; Sørensen et al. 2011). That said, no candidates for the first two enzymes of the PPP, phenylalanine ammonia-lyase (PAL; until recently) and cinnamate 4-hydroxylase (C4H; still) had been found in any streptophyte or chlorophyte algal genome (Labeeuw et al. 2015; Jiao et al. 2020; de Vries et al. 2017, 2021). A putative absence of these canonical enzymes stands in contrast to the presence of, for example, the product of C4H (*p*-coumaric acid) in a diverse set of green algae (Goiris et al. 2014). Hence, routes to *p*-coumaric acid that do not match the canonical pathway maps are likely to exist.

Our thinking about plant specialized metabolism is shaped by the means with which we can study it. For technical reasons, it is easier to measure metabolites at a global scale (metabolomics) than all enzymatic reactions that give rise to them. Yet, it is the enzymatic reactions that shape the majority of connections in a metabolic network (see also Hatzimanikatis et al. 2004). Additionally, spontaneous reactions exist and their conservation or convergent occurrences are not easily identified. This necessarily results in a metabolite-centric point-of-view when we try to disentangle metabolic networks across the green lineage on a large-scale. Yet, biochemical pathways that form the metabolic landscape are tightly regulated by signaling molecules and regulatory proteins. This heavily influences the composition and distribution of metabolites in an organism and results in a tight spatiotemporal regulation (Colinas and Goossens 2018). Hence, the regulatory pathways form a complex layer of protein–protein and protein-signal molecule interactions onto the web of specialized metabolism. Of particular evolutionary importance is that pre-existing regulatory pathways could pave the way for novel metabolites (Kliebenstein & Osbourn 2012). For example, R2R3-MYB transcription factors (TFs) are, among other factors, involved in the regulation of several specialized metabolites and it is suggested that they enable metabolic plasticity in plants (Grotewold 2005; Wu et al. 2022). Taken together, the function of a metabolite in a certain species often remains obscure. The inability to detect a metabolite under certain laboratory conditions complicates the matter: its absence often leads to misinterpretations with regard to whether a lineage has the ability to respond to particular stressors. Yet, we cannot be sure of a function of a metabolite in a species without functional studies, likewise, however, we cannot discard its presence because it was not detected in the lab. This complicates reconstruction of and inferences from the network.

As a step forward, Kliebenstein (2012) highlighted that the signaling cascades that regulate or respond to the presence of specific metabolites are more likely to be conserved across species, whereas the routes towards the metabolites may be lineage-specific at least in some steps. Given the co-expression of biosynthesis and signaling components (see discussions in Rhee and Mutwil 2014), Kliebenstein (2012) suggested to combine comparative genomics and co-expression networks to identify not only the variable routes towards one metabolite, but also the possibility of alternative metabolites that fulfill similar functions in different species. For example, the genes relevant for biosynthesis of anthocyanins is only fully conserved in vascular plants (Piatkowski et al. 2020). But recently, auronidins have been identified in liverworts and appear to function similar as anthocyanins with regard to UV-protection in angiosperms (Berland et al. 2019)—more on this below. Additionally, more and more gene clusters for a given biosynthesis pathway are identified on the chromosomes of plant genomes (Liu et al. 2020; Foflonker and Blaby-Haas 2021). These clusters can be—but are not necessarily—conserved between species and sometimes evolved convergently in unrelated species (Polturak and Osbourn 2021). Searching for such clusters can identify candidates for alternatives to enzymes that appear as missing in otherwise conserved pathways; this allows for not only relying on whether or not the full suite of ‘known’ enzymes is present, but for the identification of variations in metabolic networks and/or even the identification of a pathway de novo. For example, the biosynthetic route from cholesterol towards alpha-tomatine was identified by combining co-expression analyses and searches for gene clusters, followed by functional evidence of several of the predicted genes in the pathway (Itkin et al. 2013). In oat, cluster analyses identified a Glycosyl hydrolase family 1 required for the final glycosylation step of the saponin avenacin, whereas these reactions are usually carried out by UDP-sugar-dependent glycosyltransferase family (UGT1) in many other angiosperms (cf. Vogt and Jones 2000; Orme et al. 2019).

## Neutral evolution in the context of an overabundance of carbon

Evolution has brought forth a diversity of metabolic networks in land plants. A burst of new pathways in specialized metabolism has been associated with the origin of land plants (Weng 2014); yet, other networks appear to have deeper evolutionary routes and were modified during evolution, such as the usage of dinor-12-oxo-phytodienoic acid (dn-OPDA) and the derivative JA-Ile (Monte et al. 2018; 2019). But how did this diversity of specialized metabolism across different lineages arise? We propose that much of the metabolic diversity could have arisen by happenstance and be retained by neutral evolution; thus metabolic diversity would be the result of factors, including chance (historical contingency), fixation by happenstance (e.g. constructive neutral evolution; Gray et al. 2010; Schulz et al. 2022), and selection. This is not contradictory to the idea that for example co-evolution and arms races between host and pathogens/parasites can accelerate selection of metabolic diversity (e.g., Ehrlich and Raven 1964; Agrawal et al. 2009; Defossez et al. 2021), for which buildings blocks logically had to be already present. Yet, this scenario may appear counter-intuitive under the assumption that specialized metabolism is costly and that selective processes always result in reduction of diversity (Jones et al. 1991; Firn and Jones 2003). However, these two assumptions can be challenged.

When we consider plants and algae, carbon is, however, overabundant and specialized metabolites consist mostly of carbon. All of plant specialized metabolism benefits from carbon fixation of plants. The biological fixation of atmospheric CO_2_ is a carbon source that plants and algae need to funnel into an appropriate sink. Specialized metabolism is such a sink. To put this in numbers, the phenylpropanoid-derived wood molecule lignin (which consists of carbon, oxygen, and hydrogen) alone likely accounts for more than 15% of the biomass on land (cf. Robinson 1990; Bar-On et al. 2018). It is tempting to speculate that one of the key sinks for the earliest land plants was their rich specialized metabolism—making use of the increase in CO_2_ availability in the terrestrial habitat. On top, diverse carbon concentration mechanisms occur across the green lineage (e.g., Mackinder 2018); these mechanisms increase the efficency in photosynthetic carbon fixation by elevating the intracellular carbon levels several fold over the environmental concentration of carbon dioxide. Thus, CO_2_ availability likely resulted in the opportunity to tinker with specialized metabolism at low evolutionary constraints.

Being able to tolerate metabolic “noise” because of the low costs of fixed carbon could be a major facilitator of metabolic plasticity. This plasticity may provide an evolutionary advantage by allowing more rapid adaptation. Parasites and pathogens are expected to be strong selective factors for plant metabolic diversity (Ehrlich and Raven 1964; Agrawal et al. 2009; Kliebenstein 2012; Defossez et al. 2021). And, indeed, there is a broad range of secondary metabolites that are recurrently associated with the interaction between land plants and their pathogens: terpenes, alkaloids and phenylpropanoids, including derivatives such as flavonoids and anthocyanins (reviewed in Kliebenstein 2012). In a survey of over 400 vascular plants from grasslands in Switzerland, pathogen/parasite-pressure was one of the factors correlating with higher diversity of specialized metabolites (Defossez et al. 2021).

A new toxic compound in a plant might provide protection from a pathogen. In turn, a given pathogen isolate can by chance evolve the ability to remove this new compound or be able to detoxify a variety of compounds, providing an advantage over other isolates of the same species (e.g., Gloss et al. 2014). This results into a co-evolutionary arms race between plants and pathogens where the ability of detoxification and the appearance of new compounds can drive the metabolic diversity. Microbial pathogens and parasites that have shorter generation times than their hosts may have a distinct advantage in this: they can adapt more rapidly (de Vries et al. 2020). Yet, a study on the genus *Inga* suggests that in this case herbivores are constrained by the evolutionary defenses, particularly metabolic traits, of the long-lived plants and that these traits evolved independently and with little phylogenetic constraints (Endara et al. 2017). As such, the metabolic defense traits show rapid evolution, and Endara et al. (2017) hypothesized that, despite the longer generation times of plants, herbivores are outpaced and have to track possible host plants with a metabolic cocktail they are able to digest/detoxify. But how does lability in metabolite production arise? Enzyme promiscuity can generate metabolic noise and thus plasticity—as discussed in detail later. As such, enzyme promiscuity may allow for plants to match the evolutionary speed of their pathogens/parasites.

Let us stay for one more moment with the idea of labile evolution of defense or specialized metabolism in general. What does this mean for the evolution of metabolic networks in the context of plant terrestrialisation? It, for one, explains (among other mechanisms) the lineage-specific metabolomes observed in land plants (e.g., Defossez et al. 2021). It further emphasizes the need to include within-species information on metabolomes: Metabolic diversity will not only occur between species, but also within species. Macroevolutionary inferences on the evolution of metabolic networks and their occurrences in the earliest land plants may be biased without within-species information on its diversity and the evolutionary constraints that they experience. Indeed, different generation times or different population sizes determine how strongly drift and selection can affect the metabolic networks. For example, within-species diversity may be lower or higher depending on the algal or plant lineage and this might influence the degree of divergence of metabolic routes between species. These are important information when it comes to reconstructing the putative ancestral genetic and metabolic framework from the LCA of Zygnematophyceae (especially given their genetic diversity, see Hess et al. 2022) and land plants and the LCA of land plants. In the next section we focus on how to best disentangle those metabolic networks that have been major contributors to plant terrestrialisation and the life on land later on.

## Stressors: multi-physicochemical and multi-physiological phenomena that bear upon the routings in the web of specialized metabolism

**Fig. 2:**
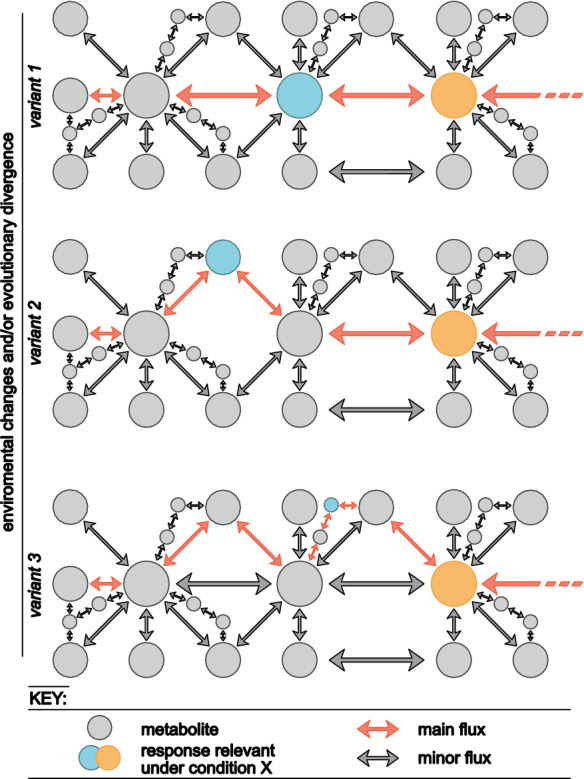
Biochemical pathways evolve dynamically. Schematic depiction of metabolic variation to allow for acclimation of a hypothetical metabolic pathway and how evolutionary processes can act upon this variation via selective or neutral evolutionary divergence (e.g., drift). The figure depicts three variants of the same pathway to indicate that (i) under certain environmental conditions the main flux can become a minor flux or vice versa. Likewise, functional compounds can shift and, under a certain condition, a metabolite from the minor flux can become a functionally relevant compound. (ii) Similarly, through evolutionary time a minor pathway producing a certain compound in one species can, for example, become the stable main flux in another species due to species-specific lifestyles. The main enzymatic route under a particular environmental condition or in a particular species is highlighted in orange (main flux), less favorable, but nonetheless occurring, enzymatic reactions are colored in grey (minor flux). Metabolites that are involved in a response to a particular condition (e.g., stress) are colored in blue and yellow. The size of the circles indicate the amount of metabolite that is produced. Here it is noteworthy, that a compound functional in a particular response does not have to be very abundant to have a functional role. Additionally, metabolites from the minor flux can theoretically be more abundant, depending on the amount of metabolization of this compound

It is conceivable that the functions of some metabolic networks bring forth properties that are adaptive in the terrestrial habitat; this is irrespective of whether these networks (and/or their wiring) arose in land plants or before, i.e. in land-dwelling algae or in aquatic algae as exaptations. To understand how evolution can shape a metabolic network we first need to look more closely at how variation can occur in (metabolic) networks. If we consider a metabolic network as a directed graph, its nodes are metabolites and its edges are reactions (enzymatic or non-enzymatic; Fig. 2). Plant metabolism spans a web of interconnected pathways that give rise to a myriad of compounds (nodes). The wires of reactions (edges) in this network have been drawn based on an evolutionary pluck and pull; the establishment of edges can be, among others, fostered by: (i) environmental conditions (e.g., reactions such as UV-induced ROS), (ii) spatial/temporal regulations (such as storage, transport, and more), (iii) evolutionary change bringing about new enzyme-coding genes through mutation, (iv) duplication (of genes or genomes) followed by neo- or sub-functionalization, (v) domain shuffling, (vi) changes in expression leading to changes in localization or time of expression of enzymes, (vii) de novo appearance of enzymes, and (viii) horizontal gene transfer. One can hence consider that the metabolic network is shaped by trial-and-error, some connections will increase the fitness of the organism, some will decrease it, and others will have no noticeable effect; some metabolites may not even have a function but represent a certain kind of metabolic “noise” that arose as a by-product of the evolutionary history of the network. That being said, evolutionary noise does not need to be a dead end. For example, a non-enzymatic chemical reaction can become biologically fixed by enzymes being recruited to catalyze it, providing further control on the process. Likewise, spontaneous reactions that occur in a reliable manner towards certain stresses may eventually become signaling components for said stressors (for discussion see Rieseberg et al. 2023).

Hence, there are considerable dynamics to the evolution of the web of specialized metabolism; several evolutionary processes shape the network of specialized metabolism of plants. Among them are neutral processes, drift, but also selective forces. With regard to plant terrestrialization, biochemical networks most often are looked at from the perspective of the role the metabolite has in extant (model) embryophytes that live on land. Yet, this might be a poor proxy for the function that said metabolite could have had in the land plant ancestor (or before). Complex biochemical networks may first emerge from a series of neutral (or slightly advantageous or deleterious but tolerable) events rather than by adaptive forces, which can then become important later on. The key idea here is that among many excess capacities that will be possible to come to existence, some receive the chance to get connected to the metabolic network. Then, due to a later event, such as loss of a pathway, change in enzyme localization, or environmental changes (e.g. higher irradiance leading to more ROS) they may contribute to the fitness of the algae or land plants. They become a lynch pin for the whole system. If the second scenario happens, the happenstance change/appearance of metabolites can provide a selective advantage and become fixed in the population. However, as long as a metabolite is not detrimental or comes at a severe cost of fitness, it is possible that the metabolite and its routes of synthesis remain existent and contribute to the metabolic background (Fig. 2). With regard to all this, the environment is the major source of selective pressures. The environment acts on the entire web of specialized pathways; yet different nodes and edges will be influenced to different degrees. Some edges and nodes will be more evolutionarily robust while others may be more labile in evolutionary terms, having the chance to arise multiple times independently.

The leverage that environmental factors have in shaping the complexity of specialized metabolic networks builds on radiation. Diversity of living organisms occurs through radiation, and so does their biological chemodiversity. It is born out of radiative processes in enzyme families; here cytochrome P450 monooxygenases are a prime example. CYP enzymes catalyze hydroxylation of diverse substrates. They are among the largest enzyme family in plants (Nelson and Werck-Reichart 2011). While some CYP subfamilies that produce compounds with essential functions appear to be under purifying selection, other enzyme clans have radiated and are shaped by gene duplications (Nelson and Werck-Reichart 2011). CYPs can be promiscuous in their substrate, as is evident from the convergent appearance of *Sm*F5H and *At*F5H (Weng et al. 2008). Indeed, high substrate promiscuity among CYPs involved in phenylpropanoid metabolism has been shown for various and divergent land plants (Alber et al. 2019). While promiscuity cannot generally be assumed to be confirmed *in planta* for all in vitro reactions (at least under a given condition), it certainly represents a large evolutionary playground where selective pressures have likely acted upon. Reducing the promiscuous space *in planta* is conceivably a driver for the occurrence of species-specific metabolic subnetworks.

Obviously, not every subnetwork is active under every environmental condition, but rather as a response to some environmental factors, which may act as stressors to algae and plants. Hence, the question is not only about the evolutionary history of the possible network space, but also about network responsiveness and subnetwork activation. A metabolic network may exist across the green lineage but different subnetworks or linear paths will be used upon stress response (Fig. 2). Here, the phytohormone salicylic acid (SA) is worth mentioning. SA can be synthesized either from isochorismate or via the routes of benzoic acid biosynthesis (Vlot et al. 2009). While in *A. thaliana* over 90% of SA is produced via the former pathway (Garcion et al. 2008; Chen et al. 2009), soybean uses both routes to a similar extent (Shine et al. 2016). Additionally, some species appear to prefer one pathway over the other depending on the stressor (Pallas et al. 1996). Investigating such multidimensional and dynamic metabolic responses that are found in the green lineage, it becomes clear that stress responses are not only a one-stressor-one-response process. Every stressor elicits a multi-physicochemical and multi-physiological phenomenon which allow for fine-tuning of the responses.

Palpable examples of metabolic flexibility are offered by carotenoids and the xanthophyll cycle (XC). The XC showcases a fine-tuned response to cover different light regimes, light condition switches, and time scales (García-Plazaola et al. 2007; Goss and Lepetit 2014). Indeed, not one XC, but up to seven XCs are currently known (Grouneva et al. 2006; García-Plazaola et al. 2007; Goss and Lepetit 2014; van den Berg and Croce 2022); some of which have been discovered in certain species or groups of organisms, while others have been reported to co-occur within one species (García-Plazaola et al. 2007). The complexity of co-occurrences may be assumed to be wider than currently known; additional XC diversity will likely be discovered as more species are investigated. In species where multiple XCs have been reported, their multi-physicochemical aspects become apparent. Owing to various physicochemical properties of the carotenoids utilized and its protein complexes, the dissipation of excess light via non-photochemical quenching (NPQ), light harvesting and photoprotection are fine-tuned by different XCs (for more, see Holt et al. 2005; Goss and Lepetit 2014; Correa-Galvis et al. 2016); further, several carotenoids, and especially XCs, strongly influence parameters like membrane stability and antioxidative activity as well as carbon concentrating mechanisms and fixation (Havaux and Niyogi 1999; van den Berg and Croce 2022). Congruently, tissue-specific differences (depending on light exposure) in the amounts of lutein (in the lutein epoxide cycle) or violaxanthin (in the well-known violaxanthin–antheraxanthin–zeaxanthin cycle) have been reported (see discussion on this by García-Plazaola et al. 2007). All of these multi-physicochemical properties influence a diversity of physiological consequences/phenotypes for plants and algae. Adding another layer to the network, many apocarotenoids derive from xanthophylls; either by spontaneous or enzymatic cleavage (recently reviewed by Moreno et al. 2021). Consequently, the diversity of XCs found across the green lineage predicts a yet unknown diversity of apocarotenoids. Given the multi-physiological roles known apocarotenoids have, we may predict that the diversity of XCs offers a pool of unknown apocarotenoid diversity that either can or has evolved into a signal impacting a physiological stress response—presenting an evolutionary hotbed for signals to emerge.

Next, we will use the PPP and its derived compounds as one of the best-studied examples of metabolic networks salient to plant terrestrialization to illustrate how all here-discussed evolutionary processes can impact the metabolic flexibility in response to (terrestrial) stressors and discuss the evolutionary history as well as resulting interconnectivity between metabolic subnetworks with regard to the emergence of new properties in this network.

## Selection on UV-associated properties and the subsequent emergence of polymers

The PPP, which is downstream of the shikimate pathway-derived phenylalanine, is a core set of routes that yields a plethora of compounds with different functions. The formative selective pressure on the PPP can easily be conceived as acting in the production of substances that ward-off abiotic and biotic stressors, among them UV-protecting sunscreens. The closest algal relatives to land plants, the Zygnematophyceae, produce phenolic compounds that likely act as important UV screens, such as the conceivably shikimate pathway-derived purpurogallins (Remias et al. 2012) and other compounds (Busch and Hess 2022). The biopolymer sporopollenin is often imbued with PPP-derived compounds that can include coumarates or flavonoids and acts as a UV-shield in land plants (e.g., Xue et al. 2020); algaenan are sporopollenin-like biopolymers that are found in diverse—but not all—chlorophyte algae (Kodner et al. 2009). Deposition of biopolymers that are akin to sporopollenin and/or algaenan were described in various streptophyte algae, including Zygnematophyceae (de Vries et al. 1983), Coleochaetophyceae (Delwiche et al. 1989), and Charophyceae (Blackmore & Barnes 1987). Recently, Permann et al. (2021) used RAMAN spectroscopy to pinpoint aromatic compounds within the brownish-colored biopolymers that surround the spores of the Zygnematophyceae *Mougeotia*. The similar localization (spores/pollen) and the presence of phenolic compounds may hint that algal sporopollenin/algaenan serves a similar UV-protecting role as it does in land plants. Additionally, UV-protecting mycosporine-like amino acids (MAAs) are detected in some streptophyte algae (reviewed in Shick and Dunlap 2002). Their biosynthetic origin is not fully elucidated, with the shikimate pathway and the pentose pathway being likely candidates (Gao and Garcia-Pichel 2011). The absorbance spectrum of MAAs covers both UVA and UVB radiations; however, klebsormidin found in different species of *Klebsormidium* and *Interfilum* from terrestrial habitats, particularly biological crusts, rather showed absorption maxima in the range of UVA radiation (324 nm; Hartmann et al. 2020). It is conceivable, that the absorption maxima of MAAs can be modulated simply due to structural alterations: that is the different amino acids that can be bound to their chromophore. This offers a conceivably simple tinkering set for the evolution of chemodiverse responses to different UV regimes.

The induction of UV-protective phenylpropanoid-derived compounds, such as flavonoids, is not only known from flowering plants (see Agati and Tattini 2010) but also from the moss *Physcomitrium patens* (Wolf et al. 2010) and the liverwort *Marchantia polymorpha* (Clayton et al. 2018). With the detection of flavonoids in the zygnematophyceaen alga *Penium margaritaceum* (Jiao et al. 2020), the deep roots of shikimate/phenylpropanoid-derived phenolics are palpable. But flavonoids are only the tip of the iceberg. In addition to the peripheral routes leading to flavonoids and anthocyanins, many routes in and derived from the PPP yield UV-protectants. Few enzymatic steps away from the core routes of the PPP, UV-protectants such as hydroxycinnamoyl-shikimates and chlorogenic acid are formed (e.g., Clé et al. 2008). Are these just mere side-effects these compounds have or was their biosynthesis under UV-stress selected for? Here, a phylodiverse view will provide important insights into the versatility that underpins their evolutionary emergence. Recently, the liverwort *Marchantia polymorpha* was shown to produce a new class of pigments called auronidins (Berland et al. 2019). These are like anthocyanins but synthesized from aurones (Berland et al. 2019). Akin to flavonoids, auronidins are likely under the control of the conserved MYB14-controlled signaling chassis for UVB protection (Albert et al. 2018; Clayton et al. 2018; Berland et al. 2019). Thus, this is a prime example where regulatory mechanisms for a similar function are conserved, but the underlying functional metabolite is different. If we take into account the likely monophyly of bryophytes (Puttick et al. 2018), we can derive that UV-response and -protection was regulated by the same regulatory network in the earliest (extinct) land plants and across its extant descendants. To infer which metabolites may have been used in the earliest land plants will be much harder to disentangle, but we have a plethora of UV-protectant metabolites that can be investigated with regard to their metabolic characteristics and evolutionary conservation when we add streptophyte algae to the picture.

Next to giving rise to UV-protectants, the PPP is arguably best known for providing backbone molecules for polymers. Most notable among the phenylpropanoid-derived polymers is lignin. The deep evolutionary origins of lignin biosynthesis are still obscure. Despite (i) the presence of deep homologs for various enzymes in the phenylpropanoid pathway even in streptophyte algae (de Vries et al 2021) and (ii) the presence of lignin-like compounds in streptophyte algae (Delwiche et al. 1989; Sørensen et al. 2011), the first emergence of polymerized lignin during the course of evolution is an open question. Understanding the deep evolutionary roots of phenylpropanoid biosynthesis can however yield important insights into key traits of land plants that go beyond their profile of specialized metabolites: Lignin is critical to our understanding of water management in the earliest land plants. Tracheophytes are not the only land plants that have water-conducting tissues. Non-vascular plants such as *P. patens* have (non-lignified) water-conducting hydroids (e.g., Ligrone et al. 2000). Here, too, TFs provide important insights into the evolution of conducting cells (see Ohthani et al. 2017); vascular and non-vascular plants appear to have conserved genetic programmes that underpin the development of transporting tissues (Woudenberg et al. 2022). NAC TFs orchestrate xylem differentiation and their concomitant lignification (Kubo 2005). Mutants of the moss *P. patens* deficient in homologous NACs showed aberrant hydroids (Xu et al. 2014)—despite that mosses do not have lignified vasculature. This signaling cascade requires the interaction as well as downstream activation of specific MYB TFs (Zhong et al. 2008; Geng et al. 2020). Several of these MYBs have control over the phenylpropanoid and/or lignin biosynthesis pathway (Liu et al. 2015). For example, MYB20, which is controlled by NAC TFs (Zhong et al. 2008; Geng et al. 2020), appears homologous to a MYB TF induced by UVB in moss (Wolf et al. 2010). As such many of these TFs that are now acting upstream of lignin biosynthesis, appear to belong to a signaling network that at least in part is 500 million years old (see Ohtani and Demura 2019). This begs the question of what is downstream of this regulatory machinery in non-vascular plants and what was downstream of it more than 500 million years ago. The example of MYB20, its conserved connection with NACs, its role in the synthesis of phenylpropanoids together with the deep routes of phenylpropanoids, it appears possible that the NAC-MYB gene regulatory network already acted on parts of phenylpropanoid biosynthesis before *bona fide* lignification evolved.

**Fig. 3:**
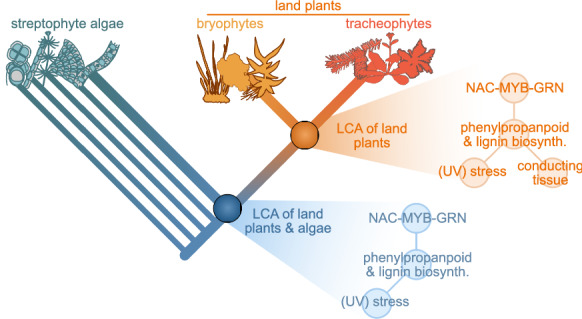
Polymers for conducting tissue born out of stress response. Evolutionary scenario for the evolution of regulatory networks that modulate the expression of plant phenolics. It is conceivable that a conserved regulatory network first orchestrated the production of diverse phenolics that ward off UV, and was subsequently extended to the usage for structural polymers that reinforce conducting tissues early in embryophyte evolution. Gene expression for highly abundant structural polymers themselves might share conserved routes with highly abundant UV shields

By considering only bryophytes and tracheophytes we can however not fully infer how likely such possibilities are. The recognition of bryophytes as a monophylum (Puttick et al. 2018) results in an evolutionary deep split between vascular and non-vascular plants. For evolutionary inferences, this means that the likelihood of any character being present or absent in the LCA is the same when it appears in bryophytes but not in tracheophytes, or vice versa. Vasculature and its lignification were assumed to be derived because of the previously assumed paraphyly of bryophytes (see also discussions in Puttick et al. 2018)—but this is not the case anymore under bryophyte monophyly. Investigating the evolutionary distribution of the molecular basis behind that trait, such as the NAC-MYB network, in streptophyte algae is important for our understanding of the evolution of routes of the lignin biosynthesis pathway tied to vasculature. This illustrates how the complexities in a metabolic network (lignin/phenylpropanoid biosynthesis) and gene regulatory networks (NAC and MYB)—which operate independent from biochemical rules—concertedly yield evolutionary flexibility (Fig. 3).

Hence, it is possible to conclude that the PPP, when first emerged, produced ‘only’ an array of specialized metabolites with properties to protect against abiotic and biotic stressors, and was later co-opted for the formation of polymers that then provided additional adaptive advantages in the terrestrial habitat. Indeed, the cuticle of the (non-vascular) moss *P. patens* is enriched in PPP-derived phenolics that are speculated to shield against UV (Renault et al. 2017). Further, analysis of the UV-resistant Antarctic moss *Ceratodon purpureus* revealed that it had nine times higher content of UV-shielding phenolics in the cell wall than in the soluble fraction (Clarke and Robinson 2008). As such, non-vascular plants appear to funnel their overabundant carbon into an array of UV-protectant compounds, whereas vascular plants use lignin formation as a primary sink (in terms of mere abundance of molecules). This makes a UV-shielding origin for the elaboration of phenylpropanoid biosynthesis and the eventual emergence of building blocks for structural polymers come full circle: It is conceivable that the earliest land plants made use of an imbuement of their cell walls with polymers (or monomers thereof) possibly first selected for their role in UV protection, but that later became recruited for structural properties.

## Conclusion

Plant specialized metabolism is tightly interwoven with plant terrestrialization and the evolution of the green lineage in general. Understanding the evolutionary history of specialized metabolic networks will enable us to decipher which roles these metabolic networks played in the earliest land plants and what has become of them in extant lineages. We have highlighted the low evolutionary constraints that photosynthesizing organisms have to play with carbon-based metabolites and the many evolutionary forces (beyond natural selection) that shape these networks. Given the metabolic plasticity and enzyme promiscuity that we see in today’s species, it can be assumed that the LCA of land plants already had a large metabolic potential, but we should also consider that the ancestral functions of some metabolites (and thus networks) might differ from that in land plants today. It is likely that many of the specialized metabolites present in the LCA and the earliest land plants have rather acted as a pool of standing variation that was later co-opted to new functions during land plant evolution. To determine at which steps during land plant evolution co-option happened, which pathways are conserved, which are convergent and what is lineage-specific, we need to embrace the diversity of non-model organisms across land plants and their close algal relatives.
